# Transmission of severe acute respiratory syndrome coronavirus 2 through asymptomatic carriers and aerosols: A major public health challenge

**DOI:** 10.1590/0037-8682-0669-2020

**Published:** 2020-12-11

**Authors:** Eduardo Tosta

**Affiliations:** 1 Professor emérito, Faculdade de Medicina, Universidade de Brasília, Brasília, DF, Brasil.

**Keywords:** SARS-CoV-2, COVID-19, Transmission, Asymptomatic carrier, Airborne transmission

## Abstract

In the absence of vaccines and effective antiviral drugs, control of the spread of coronavirus disease (Covid-19) relies mainly on the adequacy of public health resources and policies. Hence, failure to establish and implement scientifically reliable control measures may have a significant effect on the incidence of severe acute respiratory syndrome coronavirus 2 (SARS-CoV-2) infection, severity of the disease, and death toll. The average number of secondary transmissions from an infected person, or reproduction numbers (R_0_ and R), and the points at which the collective immunity begins to reduce the transmission of the infection, or herd immunity thresholds, are important epidemiological tools used in strategies of Covid-19 control, suppression, and mitigation. However, SARS-CoV-2 transmission through asymptomatic carriers and, possibly, aerosols, has been ignored, and this may affect the effectiveness of Covid-19 control strategies. Therefore, consideration of the two possible ways of transmission would substantially increase the values of reproduction numbers, but if estimates of the contingent of the population naturally resistant to the virus, plus those with pre-existing cross-immunity to SARS-CoV-2 were considered, the evaluation of herd immunity thresholds should reach their real and achievable levels.

## INTRODUCTION

A pandemic’s destructive power is determined by three factors: the virulence and infectivity of the pathogen, the degree of natural resistance and immunity of the exposed population, and the adequacy of public health resources and policies. Because it is not possible to change the characteristics of severe acute respiratory syndrome coronavirus 2 (SARS-CoV-2) and there are currently no available vaccines, the response to the coronavirus disease (COVID-19) pandemic relies mainly on the suitability of public health measures. Hence, failure to establish and implement scientifically reliable control measures may have a significant effect on the incidence of SARS-CoV-2 infection, severity of the disease, and cause of death. 

The estimation of a new pathogen’s ability to spread is a key measure in an emerging infection outbreak. The basic reproduction number (R_0_) is the main metric used to describe it. R_0_ is defined as the number of secondary cases produced by one case in a completely susceptible population in the absence of any preventive measures, while the effective reproduction number (R or Re) refers to the average number of secondary cases generated by a single index case over an infectious period in a partially immune population and under the action of preventive intervention measures^1^. Unlike R_0_, R does not assume a completely susceptible population and, consequently, will vary depending on a population’s current immune status, which will change dynamically as an outbreak event or when a vaccination campaign unfolds^2^. The epidemic is growing when R is greater than 1, it is stable if R = 1, and is reducing if R is lower than 1^1^ ^,^ ^2^ ^,^ ^3^. The two basic strategies for the containment of the COVID-19 pandemic - suppression and mitigation - make use of reproduction numbers but with different targets_._ Suppression strategies aim to keep reproduction numbers to an absolute minimum for as long as possible through quarantines and lockdowns to reduce person-to-person transmission and thereby prevent the disruption of healthcare systems. While mitigation strategies, however, do not aim at maintaining low reproductive numbers but at generating collective (herd) immunity as fast as possible by allowing controlled infection of people, and mitigating its effects^4^ ^,^ ^5^. 

The point at which the collective immunity begins to reduce the transmission of the infection is known as the herd immunity threshold. The estimated R_0_ for SARS-CoV-2 infected people was 2.5^6^, which implies that, on average, two infected individuals will spread the infection to five others during the infectious period, considering that no immunity to SARS-CoV-2 exists in the population. The herd immunity threshold is defined as 1 - 1/R_0_. Hence, in the case of an R_0_ of 2.5, the corresponding herd immunity threshold will be 0.60, or 60%, meaning that when the proportion of SARS-CoV-2 immune individuals in the population reaches this point, the infection starts to be naturally controlled. 

The concept of herd immunity threshold relies on several key assumptions, including the occurrence of homogeneous mixing of individuals within a population, that these individuals are all susceptible to infection, and that all infected individuals will develop sterilizing immunity, which will confer lifelong protection against reinfection. However, these epidemiological and immunological assumptions are not usually accomplished in real-world situations^1^ ^,^ ^2^. 

Several criticisms may be raised regarding the assumptions underlying the R_0_ and R metrics and their calculations, and hence on the levels of herd immunity thresholds of COVID-19. Two of them refer to the failure in considering the transmission of SARS-CoV-2 by asymptomatic carriers and the possibility of its occurrence in the airborne route. This failure probably gives rise to unrealistic estimates and poses doubts on the reliability of their use as important epidemiological tools of COVID-19 control strategies. 

## TRANSMISSION OF SARS-COV-2 THROUGH ASYMPTOMATIC CARRIERS

The concept of asymptomatic carriers refers to individuals who test positive for SARS-CoV-2 by polymerase chain reaction (PCR), which indicates that they are infected; thus, they represent a risk of spreading the infection^7^. The role of asymptomatic carriers in transmission poses several challenges for the control of the COVID-19 pandemic. First, since the non-infected people feel healthy, they move around more freely and are less willing to accept protective measures such as facial masks, social distancing, and quarantining. Second, as they do not present clinical manifestations of respiratory disease, they are considered safe by their social contacts. Third, because they are asymptomatic, they are usually not tested for coronavirus, and their presence and number in the community remain unknown. Finally, the acknowledgment and dimensioning of the contingent of asymptomatic carriers and the establishment of their role in the network of transmission of SARS-CoV-2 are expected to produce a tremendous impact both on R_0_ and R and, therefore, on the strategies of surveillance and control of COVID-19^8^ ^,^ ^9^.

Reliable positive PCR tests in asymptomatic individuals occur in four distinctive instances. First, the person is in the incubation period, which is the time between the moment of exposure to the pathogen and the appearance of signs and symptoms of the disease. Second, the individual has developed clinical manifestations, but has not yet had symptoms. Third, if the person turns PCR-negative later and has never experienced symptoms or signs, she should be regarded as having a subclinical disease or no disease at all due to the ability of the defense mechanisms to contain the virus. Fourth, if the individual continues to present no symptoms and the PCR remains positive for a long time, he is considered an asymptomatic chronic carrier^10^. In all these cases, individuals probably shed virus, which should be considered of epidemiological importance in control strategies. In a sample of 31 virologically confirmed asymptomatic individuals, 22 presented symptoms after hospitalization and were considered as pre-symptomatic in the incubation period, while the other nine remained asymptomatic during hospitalization. Although this latter group presented lower viral loads, the duration of viral shedding remained similar in both groups^11^, which stresses the role of asymptomatic carriers in the transmission of the infection. 

Differently from what had happened in SARS-CoV-1 infection^12^, the occurrence of asymptomatic carriers in SARS-CoV-2 infection is frequent^13^ ^-^ ^20^ and represents a considerable hindrance to the control of infection^21^ ^,^ ^22^. Based on mathematical simulations, it has been estimated that in China, 64%^7^ to 78% of infected individuals continue to be asymptomatic and undergo self-healing^20^, and they are responsible for 30% to 60% of the transmissions of SARS-CoV-2^22^ ^,^ ^23^. However, the extent of truly asymptomatic infection in the community remains to be established because no widespread PCR testing has been performed. A recent study from China that appropriately defined asymptomatic infections and followed up a group of infected individuals suggests that the proportion of infected people who never developed symptoms was 23%^24^, which is within the range revealed by a systematic review from 6% to 41%, with a pooled estimate of 16% (12%-20%)^25^. The asymptomatic carrier’s relevance in SARS-CoV-2 transmission and the ensuing imperative to contain it is well illustrated by the experience of Vo’Euganeo, a completely isolated village of nearly 3000 people in northern Italy, where the entire population was subjected to PCR testing, and all those tested positive, from 50% to 75% considered asymptomatic, were quarantined. The number of people who were sick due to COVID-19 dropped from 88 to 7 in less than 10 days^26^ ^,^ ^27^. 

An often disregarded aspect of public health policies, but with important consequences for control strategies, is the limitations of PCR-based molecular test, which is considered the gold standard for SARS-CoV-2 diagnosis^28^, especially due to the occurrence of false-negative results. It has been demonstrated that over the four days of infection before the typical symptom onset (day 5), the probability of a false-negative result in an infected person decreases from 100% (95% confidence interval [CI], 100% to 100%) on day 1 to 67% (95% CI, 27% to 94%) on day 4, and on the day of symptom onset, the median rate a false-negative result was 38% (95% CI, 18% to 65%)^29^. Such data indicate that an unknown but probably important contingent of asymptomatic and pre-symptomatic carriers escape detection by public health surveillance systems, which leads to the conclusion that the currently accepted estimates of the reproduction numbers (R_0_ and R) of the disease are inaccurate. 

## TRANSMISSION OF SARS-COV-2 THROUGH AEROSOLS

The routes of SARS-CoV-2 transmission may have an important effect on the estimation of R_0_ and R_._ Two routes have been established: respiratory droplets and contaminated surfaces. Infected respiratory droplets are expelled when an infected person coughs, sneezes, talks, laughs, or sings^30^ ^-^ ^33^. The World Health Organization has established that respiratory droplets (>5 μm) can transmit viruses only when a person is in close contact (within 1 m) with an infected person who is coughing, sneezing, talking, or singing^34^. However, studies of the physics of exhaled air and flow physics have shown that this “safety zone” is valid only if the infected person is expelling exclusively large droplets (>5 μm) during normal breathing when the velocity of the exhaled air is approximately 1 m/s^35^. However, 82% of the individuals infected by respiratory viruses exhale small infectious particles (<5 μm)^36^, which reach to a distance much farther than 1 m^35^ ^,^ ^37^. If the person coughs, the velocity of the exhaled air is 10 m/s and the droplets are expelled to a distance of more than 2 m, and if she sneezes, the velocity is approximately 50 m/s and large droplets are carried more than 6 m away^35^. Transmission may also occur indirectly through touching surfaces in the immediate environment or objects contaminated with the virus from an infected person, followed by touching the mouth, nose, or eyes^34^ ^,^ ^38^ ^-^ ^40^. A third possible route is airborne transmission, which involves the spread of an infectious agent caused by the dissemination of microscopic particles (≤5 μm) in diameter (aerosols) that remain infectious when suspended in air over long distances and time. 

The World Health Organization conceded the airborne transmission of SARS-CoV-2 in aerosol-generating procedures such as tracheal intubation^41^, although the institution has been reluctant to acknowledge this route of transmission in other situations^34^. Nevertheless, it has been firmly established as an important route of transmission of other respiratory viral infections such as those caused by influenza viruses A and B; parainfluenza 1, 2, and 3; human metapneumovirus, human rhinovirus; adenovirus; measles virus; chickenpox virus; respiratory syncytial virus; Ebola virus; MERS-CoV; and SARS-CoV^36^ ^,^ ^42^ ^-^ ^44^. Therefore, it seems unreasonable to expect that SARS-CoV-2 would be the sole exception among respiratory viruses to not be transmitted by aerosols. Indeed, it has been shown that COVID-19 patients exhale SARS-CoV-2-containing droplets into the air at an estimated rate of 10^3^-10^5^ RNA copies/min^45^. Furthermore, it has been postulated that a large proportion of the spread of COVID-19 occurs through the airborne transmission of aerosols produced by asymptomatic individuals during breathing and speaking^46^ ^,^ ^47^. The aforementioned results are based on data obtained both in experimental settings and in real-world situations. The controlled conditions in laboratory settings generate relevant, though limited data, which must necessarily be complemented by data obtained in real-world situations. It has been demonstrated that SARS-CoV-2 retained its infectivity and virion integrity for at least 16 hours in respirable-sized aerosols generated by nebulizers^48^. Moreover, viruses originating from human shedding may have an even longer survival in the environment because they are protected by components of the mucus^34^. Under experimental conditions, SARS-CoV-2 remained viable for 72 h on plastic, 48 h on stainless steel, and 24 h on cardboard^49^. 

Humans emit aerosols continuously through normal respiration^50^ ^,^ ^51^ and aerosol production increases during respiratory illnesses^47^. Although the infecting dose of SARS-CoV-2 is still unknown, it is speculated that a few hundred viral particles would be enough to cause the disease among susceptible hosts^52^ ^,^ ^53^, especially in poorly ventilated spaces, combined with low humidity and high temperature, as shown to occur with the influenza virus^54^. The infecting potential of aerosols depends on how and where they are produced. Ordinary speech aerosolizes significant quantities of respiratory particles and, for unclear reasons, certain individuals are “speech superemitters,” who emit aerosol particles of an order of magnitude of more than average, i.e., approximately 10 particles/second^55^. A 10-minute conversation with an infected, asymptomatic superemitter, who was talking in a normal volume, would yield an invisible “cloud” of approximately 6,000 aerosol particles that could potentially be inhaled by a susceptible conversational partner or others in close proximity^55^. 

It has been found that loud speech can emit thousands of droplets and aerosol particles per second, which remain for 8-14 min in a closed environment^56^, but those droplets quickly disperse in a well-ventilated room^57^. During coughing in a closed environment, the emitted large droplets rapidly fall onto the ground within 1 s, whereas aerosols will take 9 min to reach the ground when produced at a height of 160 cm (i.e., average speaking or coughing height)^57^. However, when an infected person coughs or sneezes, a cloud of pathogen-bearing particles of different sizes emerges and travels up to 7-8 m from the point of emission^58^ ^,^ ^59^. The smaller size of aerosols (≤5 μm) in comparison to that of droplets provide them with special aerodynamic features. While respiratory droplets undergo gravitational settling faster than they evaporate, thereby contaminating surfaces and leading to contact transmission, aerosols will evaporate faster than they can settle, are buoyant, and thus can be affected by air currents, which may aid in their transportation over long distances, including outside the room^46^ ^,^ ^60^ ^,^ ^61^.

Observations from real-world situations appear to corroborate the experimental data indicative of airborne transmission of SARS-CoV-2. If, on the one hand, real-world situations are closer to reality than to experimental data, on the other hand, they cannot be either provoked - due to obvious ethical reasons - or planned because their data are collected *a posteriori*. Therefore, their results should be considered as suggestive of a phenomenon and not as a confirmatory factor of its existence. Real-life situations are usually based on the detection of SARS-CoV-2 by PCR in the air and objects of hospitals where COVID-19 patients were receiving care, including patient rooms, toilets, hallways, and outdoor areas, as reporting in numerous publications^39^ ^,^ ^62^ ^-^ ^66^. Virus loads varied from 1.8 to 4.1 viral RNA copies per liter of air and were higher in air samples collected close to the infected patient^62^ ^,^ ^65^. It seems plausible to consider that prolonged permanence in contaminated settings will have a cumulative effect on the exposure to virus particles. However, these observations are limited by the fact that viral cultures were not performed in any of those studies; hence, the viability and infectivity of the virus could not be ascertained. The possibility of aerosol transmission of SARS-CoV-2 was also considered in a poorly ventilated restaurant in which infected individuals contaminated five persons seated close to them, without any direct contact or exposure to fomites^67^. The same possibility has been considered for numerous cruise ships where thousands of people aboard were infected when many of the infections occurred after the imposition of isolation that confined passengers for the majority of time to their cabins and limited direct contact, and with hand hygiene carefully followed^47^.

The possibility of airborne transmission of SARS-CoV-2 by aerosols results in several consequences for the strategies of the COVID-19 control. First, since this route is supposed to substantially increase the transmissibility of the virus, we must take this into account in the estimation of more realistic reproduction numbers (R_0_ and R), with a direct impact on the calculation of herd immunity thresholds^68^. Second, in contrast to droplets that carry viral particles that are deposited in the epithelium of the upper respiratory tract, aerosols that carry virus particles that can penetrate to the depths of the lungs, where they may be deposited in the alveoli^69^, and, supposedly, give rise to more severe disease^70^. Third, it has to be considered that aerosol-generating procedures, such as toilet flushing and, possibly, nose-blowing by infected people may spread the virus in the environment^39^ ^,^ ^62^ ^,^ ^63^ ^,^ ^70^ ^-^ ^72^. Finally, even considering that social distancing is of utmost importance for reducing the transmission of SARS-CoV-2 by virus-laden droplets, it is not realistic to maintain a safety distance of several meters, which is compatible with the demonstrated area of spreading of contaminated aerosols. This limitation stresses the importance of wearing face masks to contain SARS-CoV-2 infection. 

Face masks provide “inward” protection by filtering virus-laden respiratory particles that would otherwise be inhaled by an uninfected person and “outward” protection by trapping respiratory particles expelled by an infected person^61^. This reduces the risk of both direct and indirect viral exposure, thereby decreasing the probability of infection^46^ ^,^ ^73^. There is substantial evidence supporting the wearing of masks by the public during the COVID-19 pandemic^74^. It has been shown experimentally that face masks partially block virus-laden droplets and aerosols^75^, the latter being capable of retaining virus infectivity and integrity for at least 16 hours in the environment^48^. Indeed, face mask use results in great reduction in the risk of infection, both among healthcare workers and among the members of the community^76^ ^,^ ^77^. The filtration efficiency of face masks varies widely according to the material and the technology used for their fabrication as well as with the laboratory methodology employed to test them^78^ ^,^ ^79^. The best efficiency is achieved by disposable (or reusable under special conditions of decontamination) N95 respirators, which block, under ideal conditions, approximately 85% of particles sized less than 0.3 μm (aerosols), and 99.9% of the particles with this diameter, while surgical masks show 76% and 99.6% efficiency, respectively^79^. Reusable cloth masks have a wide range of efficiency, depending on the material used, the number of layers, and face fitness^78^ ^-^ ^81^. Considering the efficiency and the breathability, the best materials to make cloth masks are combinations of 100% cotton, nonwoven, and cotton jersey^80^. 

## DISCUSSION

Both an estimation of the contingent of asymptomatic carriers and the possible aerosol transmission must be taken into account to transform the reproduction numbers (R_0_ and R) and, hence, herd immunity thresholds, into more reliable epidemiological tools. The current policies for controlling the spread of the COVID-19 pandemic are based on the estimates of an R_0_ of approximately 2.5, a herd immunity threshold of around 60%^6^, and based on the assumption that, if no interventions were made, estimates of 7.0 billion infections and 40 million deaths are expected^82^. Mathematical simulations show that the incorporation of the contingent of asymptomatic carriers to the estimates of the reproduction numbers would tremendously inflate their values^9^, giving rise to unreachable levels of herd immunity thresholds. However, this interpretation holds a fallacy: the all individuals of the population worldwide are susceptible to SARS-CoV-2 infection at its first encounter. 

There is no completely susceptible population, neither for SARS-CoV-2 nor for any other pathogen. A variable proportion of people are not infected on exposure to the pathogen, either because they present natural resistance to it, due to their genetic background or epigenetic makeup or because they had acquired protective immunity^83^ ^-^ ^85^. In the case of novel pathogens such as SARS-CoV-2, protective immunity results from cross-protection induced by contact with related or unrelated infectious agents from the environment, or from their own microbiota^86^ ^-^ ^87^. Moreover, it should be kept in mind that susceptibility is not an all-or-nothing phenomenon but a continuum, which depends on the degree of natural resistance and/or protective immunity the person presents. It ranges from complete susceptibility to complete resistance and comprises a spectrum of intermediate states, which give rise to the different clinical presentations of the infection, ranging from asymptomatic to mild, severe, or fatal disease. 

CONCLUSION

There is an urgent need for reliable estimates of reproduction numbers and herd immunity thresholds in which transmission of SARS-CoV-2 through asymptomatic carriers and, possibly, aerosols are considered. This substantially increases the estimation of the reproduction numbers. However, if estimates of the contingent of the population naturally resistant to the virus, of those with pre-existing cross-immunity to SARS-CoV-2, which comprise 20%-50% of unexposed people^88^ ^-^ ^90^, and of the proportion of individuals presenting post-infection immunity are jointly considered, herd immunity thresholds should reach their real and achievable levels. It is possible that the current reflux of COVID-19, which is seen in several parts of the world, after a dramatic death toll, may indicate that herd immunity thresholds have been attained. However, as far as quarantines and lockdowns are relaxed, and susceptible people come into contact with the different genetic mutations of SARS-CoV-2, it is expected that new outbreaks of COVID-19 will occur until herd immunity thresholds are achieved, naturally or through vaccination. 
